# Measurement Recorder: developing a useful tool for making species descriptions that produces computable phenotypes

**DOI:** 10.1093/database/baaa079

**Published:** 2020-11-20

**Authors:** Hong Cui, Limin Zhang, Bruce Ford, Hsin-liang Chen, James A Macklin, Anton Reznicek, Julian Starr

**Affiliations:** School of Information, University of Arizona, Tucson, AZ 85705, USA; School of Information, University of Arizona, Tucson, AZ 85705, USA; Department of Biological sciences, University of Manitoba, Winnipeg, MB R3T 2N2, Canada; Curtis Laws Wilson Library, Missouri University of Science and Technology, Rolla, MO 65409, USA; Ottawa Research and Development Centre, Agriculture and Agri-Food Canada, Ottawa, ON K1A 0C6, Canada; LSA Herbarium, University of Michigan, Ann Arbor, MI 48019, USA; Department of Biology, University of Ottawa, Ottawa, ON K1N 6N5, Canada

## Abstract

To use published phenotype information in computational analyses, there have been efforts to convert descriptions of phenotype characters from human languages to ontologized statements. This postpublication curation process is not only slow and costly, it is also burdened with significant intercurator variation (including curator–author variation), due to different interpretations of a character by various individuals. This problem is inherent in any human-based intellectual activity. To address this problem, making scientific publications semantically clear (i.e. computable) by the authors at the time of publication is a critical step if we are to avoid postpublication curation. To help authors efficiently produce species phenotypes while producing computable data, we are experimenting with an author-driven ontology development approach and developing and evaluating a series of ontology-aware software modules that would create publishable species descriptions that are readily useable in scientific computations. The first software module prototype called Measurement Recorder has been developed to assist authors in defining continuous measurements and reported in this paper. Two usability studies of the software were conducted with 22 undergraduate students majoring in information science and 32 in biology. Results suggest that participants can use Measurement Recorder without training and they find it easy to use after limited practice. Participants also appreciate the semantic enhancement features. Measurement Recorder’s character reuse features facilitate character convergence among participants by 48% and have the potential to further reduce user errors in defining characters. A set of software design issues have also been identified and then corrected. Measurement Recorder enables authors to record measurements in a semantically clear manner and enriches phenotype ontology along the way. Future work includes representing the semantic data as Resource Description Framework (RDF) knowledge graphs and characterizing the division of work between authors as domain knowledge providers and ontology engineers as knowledge formalizers in this new author-driven ontology development approach.

## Introduction

Phenotypes are the set of observable characteristics of an individual resulting from the interaction of its genotype with the environment. They are paramount for describing species, studying function and understanding organismal evolution. While a large number of scientific studies and descriptions of phenotypes have been published in the taxonomic literature and are continually being published, only a fraction have been made ‘computable’ (1), an advanced state of ‘machine-actionable’. While any structured data is machine-actionable, by ‘computable’, we mean data that are unambiguously defined, can be algorithmically compared, and can be used in computational analyses in a meaningful way. The reason the vast majority of the publications involving phenotypes are not computable is that the data presented are not defined in a manner that can be used in the computation. For example, ‘perigynium beak 2 to 3 mm’ published as a text narrative is not computable: What is a ‘perigynium beak’? What does ‘2 to 3 mm’ refer to (length or width)? What landmarks were measured to obtain this value? All of these are unknown to a computer. As a result of this ambiguity, this narrative cannot be compared with an identical description in another publication, which means that considerable and in-depth curation is required to make such data useable.

Efforts have been made to convert phenotype descriptions into a computable format through a process known as ‘ontologization’, where the semantics (meaning) of a phenotype description is made explicit by translating it to formal statements consisting of terms and relations in relevant ontologies (2). In this process, human curators with domain knowledge read a phenotype description in a publication, look up matching terms in the given ontologies and write down a set of formal statements that capture the meaning of the original description as much as possible.

This practice is time-consuming and does not scale for the massive number of legacy publications, or for the millions of new publications produced each year (3). Machine learning and natural language processing techniques are being developed to speed up this process; however, for ontologizing phenotype descriptions, the success is limited due to the sophisticated subject knowledge required and complex concept translation involved in the task (1). See Table 1 for an example of translation from phenotypic characters to what is known as Entity Quality (EQ) statements. This translation leads to another critical issue of this process: intercurator variation, where, given the same description, different curators (often individuals with postdoc level of training in relevant biology areas) could come up with different translations. Intercurator variation has been widely reported in different data curation or translation settings (4–9). Similar phenomena are also well known in other intellectual activities involving human participants, for example: intercataloger variation/agreement among library catalogers and intercoder variation/agreement in content analyses used in social science studies. In the case of phenotypic character curation, a level of variation at 40% among three curators who had worked on the same project and followed the same curation guideline has been reported (10, 11). Further, a semantic gap between the consensus EQ statements among curators and the original author’s intention was found (10). Coming from different academic backgrounds, even well-trained curators can have different interpretations of the same description, choose different terms in the ontologies that seem to match the character, and/or come up with different new terms when the terms needed are missing from the ontologies. Within-project intercurator variation at this level casts serious concerns on the fitness of postpublication curations for large-scale computation or machine-based logic reasoning because curated data from multiple sources and projects will need to be combined, causing compounded, hence more serious, intercurator variation.

**Table 1.** Phenotype character description to EQ conversion

Phenotype	Entity	Quality	Related entity
Fusion of distal-carpal-1 + 2: absent	UBERON:distal carpal bone 1^a^	PATO:separated from	UBERON:distal carpal bone 2
Lateral pelvic glands: absent in males	UBERON:gland and (part_of some (BSPO:lateral region and (part_of some UBERON:pelvis and (part_of some UBERON:male organism))))^b^	PATO:absent	

UBERON, PATO and BSPO ontologies are used in these examples.

^a^ This is an example of using precomposed concept.

^b^ This is an example of postcompose a concept using existing (precomposed) concepts.

In the US National Science Foundation-funded project entitled ‘Authors in the Driver’s Seat’, we are investigating a different phenotype data and phenotype ontology production paradigm with phenotype authors in the center (12). In this paradigm, authors not only are the unmediated users of ontology, but also ‘directly’ contribute to the ontology development. While ontology engineers still play an important role, we emphasize the central position of the authors in this paradigm for a number of critical reasons. First, authors are the sole authority of the characters they discover and document. In many cases, even within a single taxonomic group, a character may not be measured in the same way (e.g. length of perigynium beak, Table 10). Hence, if the curator does not have intimate knowledge of the character, they are not going to ontologize it properly. In contrast, supporting authors to express the semantics of the characters will avoid the inaccuracies and the postpublication intercurator variation. Second, authors’ terminology usage and character formation collectively create and shape the conceptual model of a taxonomic group. A phenotype ontology covering the taxonomic group must reflect this conceptual model for it to be useful and usable ‘by the community’. In other words, phenotype ontology development needs to be ‘directly’ guided by authors’ needs when they are describing the conceptual model through their characters. Third, there are simply not enough curators to curate all historical and future phenotype publications.

**Table 10.** Task character for Task 4 with three preloaded character definitions

Task 4 character	Task 4 character’s preloaded character definitions		
Length of perigynium beak	(a) Length of perigynium beak	(b) Length of perigynium beak^a^	(c) Length of winged perigynium beak
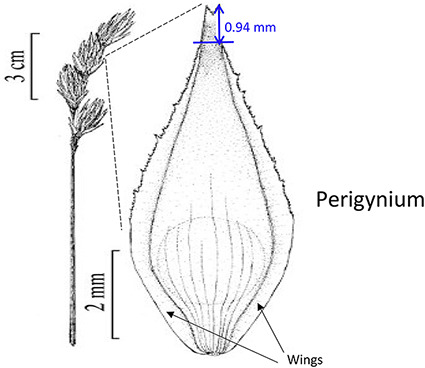	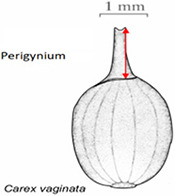	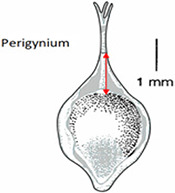	From: end of perigynium wings To: summit of perigynium Include: perigynium beak teeth

^a^ The exerted style and stigma is distracting on the illustration for this character and could be misinterpreted as beak teeth.

The overarching goal of this research is to examine the feasibility of this paradigm and eventually provide a tool to researchers creating phenotypic descriptions that will make their data more widely accessible and useable for research at large. If descriptions are semanticized and computable from the moment they are created, they would require minimal curation. This approach has the potential to eliminate a significant source of error and research costs. However, we also recognize a potential tension between data producers (e.g. taxonomists) and data consumers (e.g. computational biologists). While much of data producing work are conducted by taxonomists, the direct benefits are mostly directed at computational researchers, as some data producers may never use the data they produced in a computational setting. A key part of the ‘Authors’ project is to craft software with features that will benefit the research of the data producers and provide added value to their workflows as well. Moreover, by making their contributions more readily useable and valuable to the wider scientific community, authors may expect their work to be cited more often, a partial solution to the often stated citation impediment for the taxonomic literature (13, 14).

Our research uses phenotypic characters of the flowering plant genus *Carex* or ‘sedges’ as a model. Among plant genera, *Carex* is recognized for its exceptional diversity (>2000 species, larger than 92% of all plant vascular families (15, 16)), taxonomic difficulty and subcosmopolitan distribution. It has also become a popular model for studying speciation, ecological diversification and biogeography (e.g. (17–19)), and a large community of researchers are actively conducting taxonomic studies on the genus, including co-authors Ford, Reznicek and Starr. The combination of broad scientific interest, biological diversity and an active pool of taxonomic experts makes *Carex* an ideal model for developing and testing the new approach (also see (12) for further details).

At this time, software tools that can be used standalone or as a component in the final product are being prototyped and evaluated through usability experiments. In this paper, we describe Measurement Recorder and report the results from a set of usability studies testing the prototype of this software.

Usability refers to the degree to which a product is able or fit to be used. A software usability testing experiment examines how users interact with a software system in order to improve the effectiveness, efficiency and user satisfaction with the system. While each usability testing experiment has its own set of questions to answer, three general areas, effectiveness (how well does the system help the user complete a task), efficiency (how quickly tasks are completed) and user satisfaction (to what extend the user is satisfied with the system), are covered more or less by all usability experiments (20). For example, in recent years, DataOne (The Data Observation Network for Earth) has conducted several usability studies on its research data services (21).

Two software usability experiments involving three groups of participants are reported in this paper. Collectively, our findings answer the following questions corresponding to the design goals of the software:

- Functionality (effectiveness and efficiency): Are users able to use this software to define characters and add terms to phenotype ontology without training?
- User adoption factor: Do users appreciate Measurement Recorder for its data quality control functionality?
- User adoption factor: Do users appreciate Measurement Recorder’s support for character illustrations and character reuse?
- Data quality effects (effectiveness): Does shared access to measurement characters encourage definition convergence among users?
- Data quality side effects (effectiveness): Does shared access to measurement characters result in users accidentally using an inappropriate character?

This paper is organized as follows. We start by describing Measurement Recorder prototype and then introduce the experiment design in the Method section. Relevant results from all the experiments are then presented around the five research questions in the Results and Analyses section, where the resultant improvements on the software itself are also reported. Following a discussion of our semantic-based approach, we end the paper with plans for future research.

## Methods

### Design goals of Measurement Recorder

The accurate and unambiguous measurement of phenotypic characters is essential to biology and other disciplines. Phenotype characters fall into two broad categories. Continuous characters are those based on numeric measurements, such as lengths, widths or ratios. There are an infinite number of values between any two continuous values, and the values are related to each other in a logical order (lowest to highest). On the other hand, categorical characters have a finite number of values that may or may not have a logical order (e.g. number of flowers, colors or surface textures). The need for a semantics-aware Measurement Recorder was raised by systematist stakeholders at the first project advisory meeting. With the goal of building a semantics-aware phenotype character recorder covering both continuous and categorical characters, we started with the development and test of Measurement Recorder for continuous measurements as the first module. The lessons learned will lead to a better design of the module and also inform the design of the final system tentatively named ‘Character Recorder’. The specific design goals for Measurement Recorder include the following:

- Assist users, mainly biologists, to define characters and record measurements in a semantically clear manner;
- document the usages of the defined characters within a domain (e.g. the plant genus *Carex*);
- encourage reuse and convergence on character definitions among users;
- support features, such as character illustrations, to incentivize user adoption of the application and
- be easy to use without training.

The design goals were informed by the target user profile we developed through interviews of the biologists on this (3) and other projects (2). The profile of the target user groups is described as: professionals and students in the biological sciences (e.g. taxonomists or other evolutionary biologists) who need to document phenotypes for their research or work in general. While they use analytical software and spreadsheets (e.g. Excel) in their work, they do not have the time or the inclination to navigate complex software systems to complete routine tasks such as documenting measurements. This profile is additionally confirmed by a recent survey we conducted with 97 self-selected biologists who work with phenotype data: 6% of responders indicated that they do not wish to spend any additional effort (more than their current workflow) to make their data computable, while 35% said they are only willing to spend up to 5% more effort making their data computable. Further, 43% of the responders indicated that they are not eager to use a controlled vocabulary/ontology if using it is not mandatory (which is the case now). Although the survey also shows that over 50% responders are willing to take on > 5% effort and use a controlled vocabulary voluntarily, we choose to avoid designs that are dramatically different from the existing tools to encourage adoption by as many users as possible (22). Thus, while introducing novel semantics-checking features, we maintained a familiar spreadsheet-based user interface to alleviate the initial learning curve. Other projects, notably TaxonWorks and Morph∙D∙Base, both reviewed later, are exploring novel designs.

In designing Measurement Recorder, the methodology established in (24) was adopted. First, a conceptual model was established, taking into consideration the user profile and the user tasks (organism character recording). The conceptual model was then mapped to functions or features that needed to be implemented in the software. Several versions of user interface sketches were drafted and evaluated by the designers before one version was selected and implemented.

The key difference between Measurement Recorder and an Excel spreadsheet lies in the former requiring a character (e.g. length of perigynium beak) to be defined and saved in the phenotype ontology before it can be used to record measurements. While a user is defining a character, Measurement Recorder checks the terms used and adds new terms to the ontology, so the complete information about the character is recorded. It also allows users to reuse characters defined by others to encourage convergence on character usages.

The key difference between other approaches and the author-driven ontology development approach experimented in our research is that in our approach, regardless of the custodian, the ontology is open to the authors and the authors can add terms (including characters and their definitions) to the ontology when they need them, while in other approaches, the ontology is closed to the authors and authors’ terms must go through a (often lengthy) vetting process before being added or rejected. The reasons that we believe our approach is needed for sustainable computable data production are elaborated in Discussion.

### User scenarios of the Measurement Recorder

**Figure 1.**
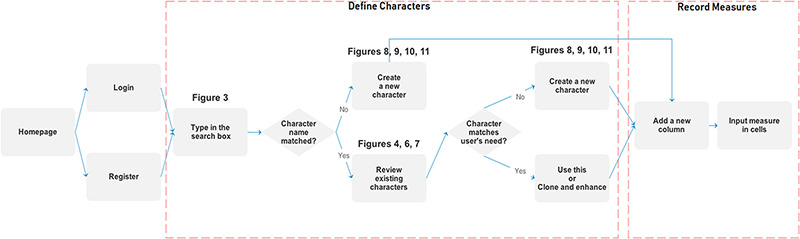
Two main workflows supported by Measurement Recorder.

Figure 1 illustrates the workflow supported by the Measurement Recorder and describes two usage scenarios: (i) the user reuses an existing character and (ii) the user creates and defines a new character. Relevant screen captures are marked in the illustrations and presented next.

#### Reuse an existing character

**Figure 2.**
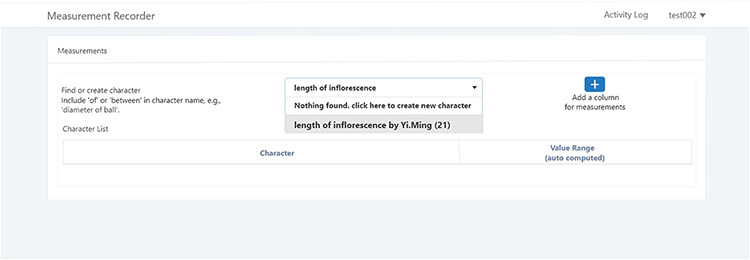
Search/create a character function of Measurement Recorder.

**Figure 3.**
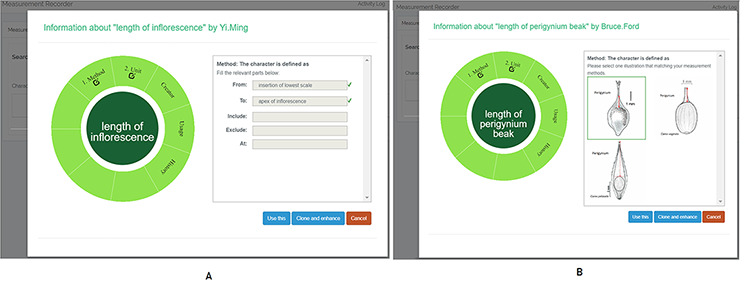
Character information consists of measurement method, unit, creator, usage and history information in Measurement Recorder. (A) The Method form is used to enter or display a definition for the character. (B) Alternatively, if available in the system ontology, an image is displayed to illustrate how the measurement is done.

**Figure 4.**
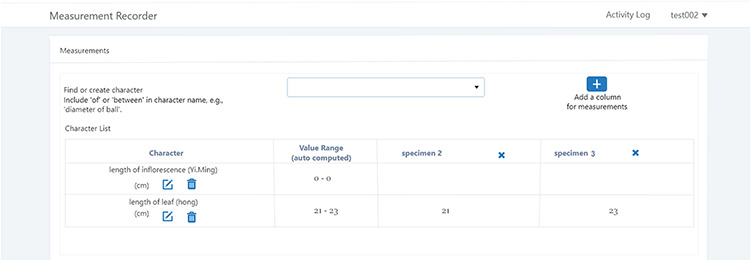
Table view of the measurements in Measurement recorder.

**Figure 5.**
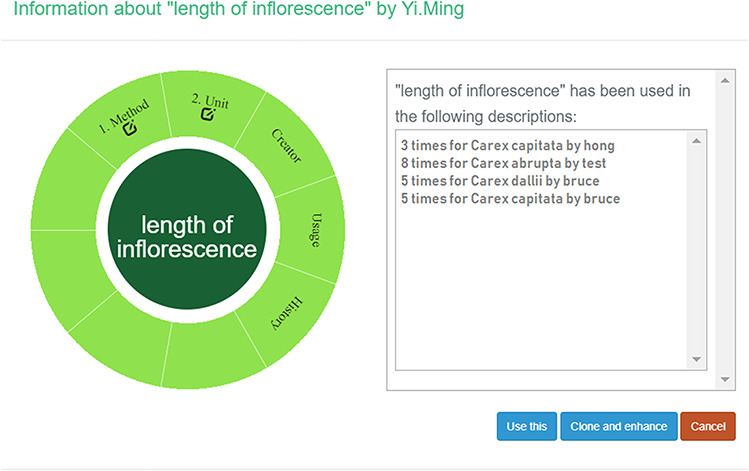
Character usage information in Measurement Recorder.

A taxonomist is preparing a taxonomic revision based on physical specimens. They plan to measure a set of phenotypic characters on these specimens. They log into the system for their taxonomic group, and type in a character name or phrase (e.g. length of inflorescence) that they are planning to measure. The system shows the existing characters matching ‘length of inflorescence’ with the creator’s names and the usage counts (Figure 2). They recognize a character created by a trusted colleague so they open that character and scan through its measurement method definition (Figure 3). The definition may be displayed as a form, or as one or more illustrations if available. The green check marks by the definition entries indicate that the terms entered are known to the ontology (Figure 3A). The user finds that the measurement method is exactly how they would measure inflorescence length, so they click on the ‘Use this’ button (Figure 3) to reuse this character. The character is loaded into their spreadsheet and they start to enter measurement values for their set of specimens (Figure 4). The new usage count of this character is updated by the system in the background (Figure 5).

The user can also enhance an existing character (e.g. improve the definition) and then use it. The ‘Clone and enhance’ (C&E) button supports this usage. Enhanced characters are expected to be ‘semantically equivalent’ to the existing character, in the same sense as in ‘equivalent classes’ in OWL, the Web Ontology Language. Two equivalent classes have exactly the same set of members. Although the definitions may be represented differently between the original and the enhanced versions, they should refer to the same measurement. With the enhancement, the current user will have shared creatorship with the original creator of the character. Technically, the enhanced character is treated as a new character and is added to the ontology as equivalent to the existing character. In the experiments, we used a home-grown CAREX ontology developed in consultation with the Plant Ontology Consortium (http://planteome.org/).

#### Create a new character

**Figure 6.**
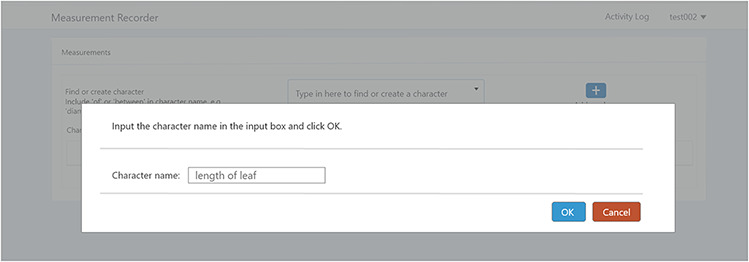
Provide character name to create a new character in Measurement Recorder.

**Figure 7.**
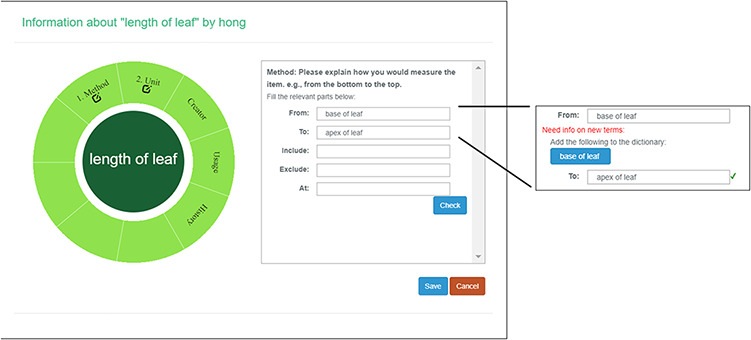
Measurement method and semantic check in Measurement Recorder. When check button is clicked, the input form may extend to show ‘need info on new terms’ if some terms entered are new to the ontology. The center circle in the left green wheel will turn yellow to indicate that this character is being updated. Method segment in the wheel will turn dark green to indicate method definition is checked.

**Figure 8.**
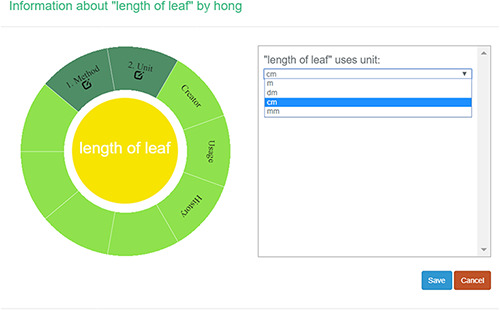
Measurement unit information in Measurement Recorder.

A taxonomist is preparing a taxonomic revision, and they plan on taking morphological measurements on a set of specimens. They log into the system for their taxonomic group, and type in the character (e.g. length of leaf) that they intend to measure in the Search/Create a Character box (Figure 2). The system finds no existing characters matching their character and offers an option to create a new character (Figure 6). The system presents a measurement method definition form, and they enter the landmarks that start and end a measurement. (Figure 7, left side). They will check if the landmark terms they entered are in the ontology. If not, they will have a chance to add and define these terms (Figure 7, right side). Next, they select a unit of measurement (Figure 8). After saving the character, the character is loaded into the spreadsheet and they start to enter measurements for the specimens under study (Figure 4). Behind the scene, the software adds the new landmark terms as subclasses of ‘CAREX: anatomic structure’ and the new character as a subclass of ‘CAREX: perceived quality’ in the CAREX ontology, where CAREX:anatomic structure is equivalent to UBERON:anatomic entity (http://uberon.github.io/) and CAREX:perceived quality is a subclass of ‘PATO:quality’ (http://www.obofoundry.org/ontology/pato.html). In the meanwhile, the system increases the usage count of the character by one.

### Usability study experiment design and subjects

Like many other usability studies in information science, we recruited university students to participate in our experiments. This is because recruiting a large number of professional biologists for usability tests for each module and prototype is not feasible. Moreover, by testing the software on students from different disciplines (information and biology), we can explore issues related to usability versus those related to biological knowledge. Last, biology students are potential users of the software. Exposing them to data quality issues, and obtaining feedback from them, not only helps to improve software design, but also provides the students with training in how to collect data for primary research. Usability tests on the final product, Character Recorder, will involve professional biologists.

In the experiments reported in this paper, participants used Measurement Recorder to complete a set of tasks that involved defining how a character should be measured in words and recording values for such characters for a set of objects (e.g. a leaf). Each task was presented on a sheet of paper, where the object and how it should be measured were graphically illustrated. The participants’ interaction with the software and their responses to a questionnaire were automatically logged by the computer. The study was approved by Human Subject Research review authorities at the University of Arizona and the University of Manitoba.

**Figure 9.**
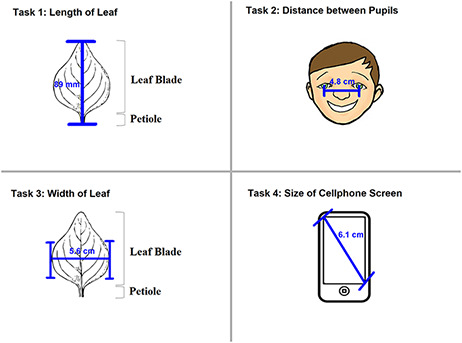
The four measuring tasks used in the Individual Experiment.

Two experiments, named ‘Individual’ and ‘Shared’, were carried out from September to November 2018 in this order. The Individual Experiment was designed to evaluate the general usability of the software. Twenty-four participants were recruited from the undergraduate programs of the School of Information at the University of Arizona for this experiment. The tasks involved measuring common objects, shown in Figure 9. The researchers manipulated the CAREX ontology for this experiment by including a different number of useful terms or character illustrations to make the tasks more or less difficult: (i) length of leaf: the ontology contained one illustration that matched the task illustration and two nonmatching ones; (ii) distance between pupils: the ontology contained many terms useful for defining this character, but no illustrations were provided; (iii) width of leaf: the ontology contained some useful terms, but no illustrations were provided and (iv) cellphone screen size: the system ontology contained no terms needed to describe this character, and no illustrations. In the Individual Experiment, participants were asked to ignore characters created by other participants, hence the name ‘Individual’. A random half of the participants were given the tasks in the order given above and shown in Figure 9 (known to the researchers as the easiest to the hardest, or ‘easy-order’, while the other half completed the tasks in the reversed order or ‘hard-order’). Depending on participants’ availability, they completed the experiment in batches of 1 to 4 participants at a time in a cubicalized computer laboratory.

**Figure 10.**
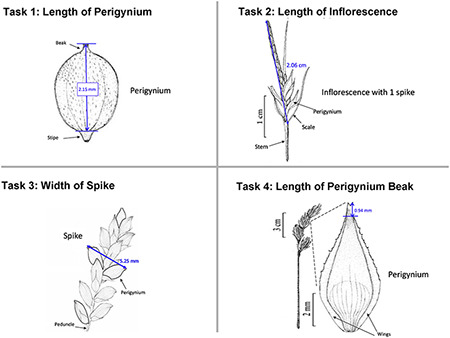
The four measuring tasks used in the Shared Experiment. Illustrations modified from the *Carex* treatments in the Flora of North America, Volume 23, pp. 389, 560, 457 and 357. Illustrations by Susan Reznicek (23).

In addition to the general usability of the software, the Shared Experiment was designed to evaluate software data quality features for users with different levels of subject knowledge. User tasks in the Shared Experiment, as shown in Figure 10, involved technical measurements of plant structures (e.g. the width of the inflorescence of some *Carex* species). Participants were asked to define their task characters on paper independently before using Measurement Recorder. Figure 7 shows the set of five fields implemented in the software and the same set was used in the paper-based exercise. These fields were obtained through a manual content analysis of the Measurements sections of several published *Carex* descriptions and approved by the *Carex* experts on the team. When using Measurement Recorder, participants were encouraged to reuse characters created by others, including the six characters that were preloaded into the system. All preloaded characters were created by a co-author who is a *Carex* expert and were made different in terms of their number of usages (20 vs. a few times), displayed creator names (Bruce Ford, known to the participants, vs. Yin Ming, a made-up name), number of illustrations in the system (ranging from 0 to 2, with some illustrations as distractors), and whether they matched a task character (not all preloaded characters matched a task character). See Appendix 1 for details. Unlike the Individual Experiment, the measurement tasks were not manipulated to create different levels of difficulty and were given to all participants in a fixed order as shown in Figure 10. Information attached to preloaded characters (usage counts and creators) were meant to reveal factors that may affect a user’s decision of reusing a preloaded character.

Participants in the Shared Experiment were recruited from two different undergraduate classes at the University of Manitoba. One, a second-year class, had a general knowledge of flowering plant structures, but not specifically *Carex*. A third-year class had a more in-depth knowledge of plant structures, including *Carex*. In addition, the third-year class had experience in using taxonomic descriptions and keys in the identification of plants. We shall refer to the first group as ‘shared-NS’ (nonexpert participants) and the second group as ‘shared-ES’ (expert participants) hereafter. Shared-ES and shared-NS groups completed the same set of tasks given in the same order in the same computer laboratory, with the shared-NS group following the exercise immediately after the shared-ES group.

One questionnaire was developed for each of the Individual/Shared Experiments after consulting various software usability questionnaires listed at http://edutech-wiki.unige.ch/en/Usability_and_user_experience_surveys. These questionnaires all have established validity and reliability. We borrowed heavily from the two Unified Theory on Acceptance and Use of Technology (UTAUT) questionnaires: the original questionnaire published in 2003 and a 2014 version with cross-cultural validation (25, 26). UTAUT models technology acceptance with dimensions include ‘performance expectancy’, ‘effort expectancy’, ‘attitude toward using technology’, ‘social influence’, ‘facilitating conditions’, ‘self-efficacy’, ‘anxiety’ and ‘behavioral intention to use the system’. Each dimension includes 3 to 4 questions.

Since Measurement Recorder was just a prototype, questions in the dimensions ‘social influence’ and ‘behavioral intention to use the system’ were not relevant. From each of the remaining dimensions, we selected one question targeting our research goals. For the questions not covered by UTAUT, we borrowed and modified questions from other sources listed in the link above. For example, one question was modified from the original ‘I have the “knowledge” necessary to use the system’ (UTAUT) to ‘I have the “skills” necessary to use the system’. The modification was necessary because we would like to know if the software itself was intuitive to use, not to test if the undergraduate students had sufficient biology knowledge to make the best use of the system. The goal was to use a minimal set of questions to solicit participant opinions to answer the set of research questions. Some questions were shared between questionnaires and others were unique. Mixed positive questions (‘feature X is helpful’) and negative questions (‘feature X is not helpful’) were used to avoid user bias. Please note that responses provided by the participants are not the sole source of data we analyzed: user-system interaction logs and paper/machine-based characters created by participants provided additional sources of data to validate our findings. All materials used in the experiments are included in Appendices 1 to 3.

Before the Individual Experiment was carried out, five pilot studies were conducted to identify major issues with the experiment materials and instructions. A few issues were identified and fixed before the experiments started. For example, a survey question was modified from ‘I find Measurement Recorder difficult to use’ to ‘I find Measurement Recorder difficult to use at the beginning’. Some pilot participants found the original question difficult to answer as the exercise became easy after completing the first task. The concept of semantic-clear measurements was also foreign to pilot participants: several noting that they thought the software would take measurements automatically off an image. Because of this, a short introduction video on the motivation for developing Measurement Recorder was made for the participants to view before starting tasks. The video for the Individual Experiment was 3 min 43 sec long and featured the same example of ‘perigynium beak’ used in the Introduction to this paper and a brief overview of Measurement Recorder. The video for the Shared Experiment was 9 min 4 sec long and also included a review of some *Carex* morphological structure terms and a more detailed description of the software.

In summary, the basic procedure for a session for both the Individual and Shared Experiments was:

- watch the introduction video;
- complete the measurement tasks in a given order and
- complete the questionnaire relevant to the experiment.

There was no training conducted on the use of the software for the participants, but the video was accessible to participants throughout the session. This setting simulates the reality when the final software is available online for new users to learn and use.

All participants received $10 for their participation, regardless of the experiment they participated in. Some participants also received a course assignment credit offered by their instructors, who were not involved in the study beyond making an announcement to recruit the participants. All participants have >1 year of undergraduate study and live in the United States or Canada.

## Results and analyses

Twenty-two of the 24 participants in the Individual Experiment completed all 4 tasks, and their data are analyzed below. Among these, 10 completed the tasks in the easy order and 12 in the hard order. Data from the other two participants were excluded from the analyses because they failed to follow the experiment instructions.

Thirty-two of the 34 participants completed the entire session in the Shared Experiment. Among these, 19 were in the shared-ES group and 13 were in the shared-NS group. Two participants in shared-NS group did not complete the tasks due to system overload (slow server responses).

Data collected from responses to the questionnaires and from the system user logs were analyzed to answer the set of research questions.

Survey questions with the five-point Likert scale (i.e. strongly agree, agree, neither agree nor disagree, disagree, and strongly disagree) are scored using (i) agree percentage, which is the percentage of the participants who selects ‘agree’ or ‘strongly agree’ and (ii) mean score and standard deviation, where a ‘strongly agree’ scores 5, and ‘strongly disagree’ scores 1. A score of 3 indicates ‘neither agree nor disagree’.

### Research Question 1: Is Measurement Recorder intuitive enough to be used without training?

Survey questions relevant to this research question and their scores are presented and grouped into ‘easy to use’ indicators and ‘hard to use’ indicators in Table 2. Responses from both Individual and Shared Experiments were largely consistent. The data showed that participants had the skills to use Measurement Recorder (mean agreement scores = 4.18, 4.13) and that it became easier to use after some practice (mean agreement scores = 3.86, 4.13). Participants also felt that having more built-in assistance would make the tool more effective (mean agreement scores = 4.00, 4.09), although they did not feel apprehensive when using the tool on its own (scores = 3, indicating neutral). Additionally, the Shared Experiment (biology) participants found Measurement Recorder easier to learn and use than the Individual Experiment (information science) participants (see Questions 3 and 5 in Table 2). This may be attributed to the longer introduction video viewed by the Shared Experiment participants and suggests a need to include a video demonstration of the software on the website hosting the final product.

**Table 2.** Survey questions and their agreement scores related to Research Question 1: use without training

Category	Questions	Agree		Mean score (SD)	
		Individual EXP (N = 22)	Shared EXP (N = 32)	Individual EXP (N = 22)	Shared EXP (N = 32)
Easy to use indicator	(1) I have the skills necessary to use Measurement Recorder.	77%	81%	4.18 (1.006)	4.13 (0.871)
	(2) Figuring out how to operate Measurement Recorder is easy for me.	73%	63%	3.77 (1.152)	3.50 (1.078)
	(3) I find Measurement Recorder is easy to use after some practice.	77%	81%	3.86 (0.941)	4.13 (0.871)
Hard to use indicator	(4) I feel apprehensive about using Measurement Recorder.	36%	44%	3.09 (0.971)	3.09 (0.780)
	(5) I find Measurement Recorder difficult to use at the beginning.	68%	47%	3.91 (1.192)	3.28 (1.780)
	(6) I could complete a task more effectively using Measurement Record if there were more help built into the system.	64%	78%	4.00 (0.926)	4.09 (0.818)

**Figure 11.**
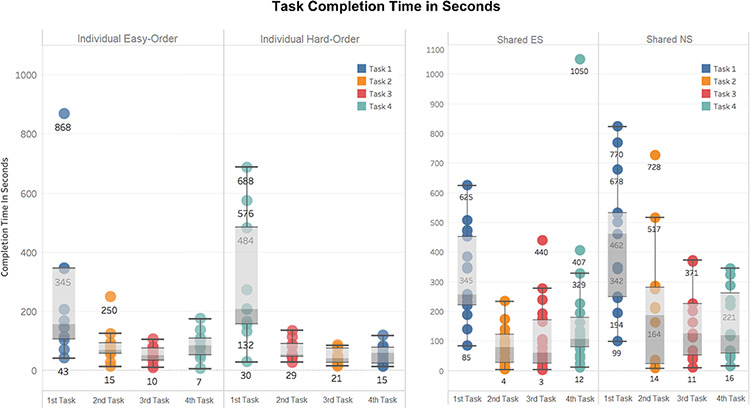
Completion time in seconds for each task. Left: Individual Experiment, showing easy/hard orders. Right: Shared Experiment, showing ES/NS groups. Different colors indicate Tasks 1 to 4 as shown in Figures 9 and 10. Labels on the x-axes here indicate task completion order: first task is the first task participants completed in their experiment.

This set of self-reported scores indicates that the software can be used without training, although more built-in help is desired. To verify this finding, task completion times were extracted from the system’s user logs and shown in Figure 11. Statistically significant differences were found between the task completion times for the first and second tasks in each of the experiment settings: Individual Experiment’s easy and hard orders, and Shared Experiment’s shared-ES and shared-NS groups (Welch Two Sample *t*-test, *P*-value < 0.001). No significant differences were found in task completion time among Tasks 2, 3 and 4 in all experimental settings. In other words, regardless of perceived difficulty levels of the four tasks, the initial task was always the most time consuming, and all latter tasks took about the same amount of time. A significant difference in the average task completion time between the shared-NS and shared-ES groups was found at a 95% confidence level (*P*-value = 0.022, Figure 11, right). This may be partially attributed to a software overload experienced by the shared-NS group.

The median completion times for Task 1 were 2 min 30 sec for Individual easy-order, 3 min 20 sec for Individual hard order, 4 min 15 sec for shared-ES and 7 min 30 sec for shared-NS. With participants in the shared-ES group most resembling our target users, professional biologists, the median task completion time assessed for Task 1 confirms that the system meets the design target: users should be able to figure out how to use Measurement Recorder on their first encounter within 5 min, without help.

Additional user interaction log analyses were conducted to identify frequent user errors: (i) not formulating character names by using ‘of’ as required by the system (accept ‘width of leaf’ but not ‘leaf width’) and (ii) attempting to save a character without selecting a unit. The error counts were positively correlated with task completion times for the Individual Experiment (Pearson correlation coefficient = 0.53, *P* < 0.05). These errors occurred less frequently among biology participants and were not correlated to task completion time for the Shared Experiment. This finding suggests that existing instruction and error messages did not adequately capture the users’ attention. The system has been redesigned to provide more help and prevent the occurrence of such errors (see Software improvements below).

One key and novel feature of Measurement Recorder is the ‘need info on new terms’ (Figure 7, right side), where users are asked to add a term (and provide a definition) that is not in the ontology when defining a character. Table 3 shows the participants’ survey responses related to their reaction to this feature. These data show that a majority of the participants (59%) understood the intention of the feature, and even more so (75%) if they were biology (i.e. Shared Experiment) students. The distributions based on the self-reported data matches what is presented in the user logs.

**Table 3.** Counts of answers to ‘My reaction to “need info on new terms” while completing the task was’

Question (My reaction to ‘need info on new terms’ while completing the task was)	I did not notice ‘need info on new terms’	I noticed it and added terms to the dictionary	I noticed it but did not know what to do with it	I noticed it but chose not to act on it
Individual experiment (N = 22)	Question not included.	13 (59%)	7 (32%)	2 (9%)
Shared experiment (N = 32)	1 (3%)	24 (75%)	7 (22%)	0

These findings provide a positive answer to Research Question 1: for a majority of the users, Measurement Recorder is intuitive to use without training, but there is more that can be done to further improve this prototype. For example, provide help information on the feature ‘adding terms to the dictionary/ontology’.

### Research Question 2: Do the users appreciate Measurement Recorder for its data quality control functionality?

Survey questions related to Research Question 2 and their agreement scores are presented in Table 4. Because participants in the Shared Experiment are biology majors, a set of questions closely related to biological data quality, which were not used in the Individual Experiment, were included in the Shared Experiment questionnaire. Questions that were not included in the questionnaire for a specific experiment have a score of ‘N/A’ in the table.

**Table 4.** Survey questions and their agreement scores related to Research Question 2: user appreciation

Category	Questions	Agree		Mean Score (SD)	
		Individual EXP (N = 22)	Shared EXP (N = 32)	Individual EXP (N = 22)	Shared EXP (N = 32)
Data quality control	(1) I find Measurement Recorder useful for explaining how a measurement is taken.	68%	97%	3.818 (1.140)	4.281 (0.634)
	(2) I think Measurement Recorder could be a useful tool for teaching biological measurements.	N/A	88%	N/A	3.844 (0.448)
	(3) Using Measurement Recorder is NOT a good idea for data quality control.	9%	N/A	2.5 (0.802)	N/A
	(4) Using Measurement Recorder is NOT a good idea for improving consistency among different researchers.	N/A	0%	N/A	1.813 (0.535)
	(5) Measurement Recorder will help increase the accuracy of measurements in scientific publications.	68%	97%	3.864 (0.834)	4.313 (0.535)
				
Compare to excel	(6) If a task requires measurement definitions, I would prefer Excel over Measurement Recorder.	36%	N/A	3.318 (1.129)	N/A
	(7) If a task requires measurement definitions (explaining how a measurement is taken), I would prefer Measurement Recorder over Excel.	N/A	88%	N/A	4.156 (0.808)
	(8) Using Measurement Recorder enables me to accomplish the measurement takes more accurately than using Excel.	55%	84%	3.545 (1.057)	4.188 (0.780)
User experience	(9) Measurement Recorder makes data recording more interesting.	55%	56%	3.409 (1.054)	3.625 (0.793)

The findings from the Individual Experiment and the Shared Experiment are consistent with this research question. These data show a relatively strong agreement that Measurement Recorder would help improve data quality in terms of accuracy and consistency (Questions 1, 2, 3, 4 and 5). Participants, especially biology participants, agree that it is preferable to Excel for data quality control (Questions 6, 7 and 8) and that it does not make data recording more boring (Questions 9). In addition, biology participants strongly agree that Measurement Recorder could be a useful tool for teaching students how to gather biological measurements.

### Research Question 3: Do the users appreciate the support for illustrations and character reuse?

The set of questions related to Research Question 3 and their agreement scores are shown in Table 5. Again, ‘N/A’ indicates a question was not included in the questionnaire for a specific experiment.

**Table 5.** Survey questions and their agreement scores related to Research Question 3: illustrations and character reuse

Questions	Agree		Mean Score (SD)	
	Individual EXP (N = 22)	Shared EXP (N = 32)	Individual EXP (N = 22)	Shared EXP (N = 32)
(1) Measurement illustrations shown in the ‘Method’ section make my work more efficient.	73%	91%	3.905 (0.995)	4.500 (0.672)
(2) I was worried that my measurement definitions may not be the best.	68%	78%	3.682 (0.995)	3.969 (0.822)
(3) Writing definitions for terms was difficult for me.	32%	41%	3.045 (1.090)	3.219 (1.211)
(4) If allowed to view the definitions created by other users, I would feel more comfortable using Measurement Recorder.	77%	N/A	3.818 (0.795)	N/A
(5) I used ‘C&E’ function to create my character.	N/A	20 [YES] 12 [NO]		
(If 5 = YES) ‘C&E’ makes it quicker to create a character I needed	N/A	95% [19/20]	N/A	4.600 (0.754)

As shown in Table 5, responses to definition related questions (Questions 2 and 3) indicate a relatively strong level of agreement that participants were not confident in their definitions for the characters, even though they might not perceive writing the definition as a difficult task (Question 3). Interestingly, more biology participants (i.e. Shared Experiment) found it difficult to write definitions (Question 3), suggesting that increased knowledge made them more aware of the underlying complexity of the required task.

There was strong agreement among participants on the usefulness of illustrations for understanding character definitions, with a stronger agreement in biology participants (mean agreement score of biology participant = 4.5, 91% participants agree). A Fisher’s Exact Test was performed but failed to find significant correlation between ‘like illustrations’ and ‘difficulty with definitions’ (*P* = 0.389). This suggests that participants with no difficulty in writing definitions also found illustrations helpful.

Although participants in the Individual Experiment were instructed not to use others’ characters, the user interaction log shows that 20 out of 88 total characters were created through reusing characters created by others via methods ‘Use this’ or ‘C&E’. Seventy-seven percent of all participants expressed a desire to view characters created by others (Table 5, Question 4).

Participants in the Shared Experiment were encouraged to review and reuse existing characters if needed. In each group (shared-NS and shared-ES), all participants noticed the relevant characters created by others, and a vast majority checked characters created by others (for detailed analysis, see Research Question 5). Twenty out of 32 participants reused some characters created by others via ‘C&E’ and found the feature increased efficiency (Table 5, Question 5, agreement score = 4.6). From this, we can infer that the other way of reusing characters, ‘Use this’, would have a similar effect because the two mechanisms are very similar, and ‘Use this’ requires fewer user actions.

Table 6 shows the factors that influence character reuse decisions as reported by the 23 Shared Experiment participants that reused characters by ‘Use this’ or ‘C&E’.

**Table 6.** Factors influenced user decisions on reusing a character created by others in Shared Experiment

My decision on whether to adopt a character created by others was influenced by the following factors (N = 23)	Strong influence	Some influence	Weak influence	No influence	Overall strength^a^
Meaningfulness of its method definition	15	6	2	0	59.0
Appropriateness of its unit	12	6	3	2	51.0
Frequency of its usage	8	8	4	3	44.0
Reputation of its creator	7	7	1	8	36.0
Change of history of the character	0	5	4	14	14.0
Others	0	3	3	17	9.0

^a^ Overall strength of influence is a weighted sum of different levels of influences and it = 3 × number of ‘Strong influence’ + 2 × number of ‘Some influence’ + 1 × number of ‘Weak influence’ + 0 × number of ‘No influence’.

The most influential factors are meaningfulness of method definition, appropriateness of unit, followed by frequency of usage and reputation of creator. The change of history of the character has weakest influence. This finding supports the design decision of including character usage and creator information beside the character name in the character selection dropdown list. A few participants selected an unspecified ‘others’ factor, but none as a strong influence factor, which suggests that all major factors are captured and presented in the system.

### Research Question 4: Does shared access to measurement characters encourage definition convergence among users?

In this section we test the hypothesis that allowing the users to access others’ character definitions through the ‘Use this’ and ‘C&E’ features would reduce variation and promote convergence in user characters.

First, the survey responses from the 20 participants in the Shared Experiment who used the ‘C&E’ feature were examined. These data showed that 19 out of the 20 participants agreed that this feature made it faster to create a character they needed, while 16 out of 20 believed it made their characters more consistent with others’ characters.

Then, character definitions recorded on paper were compared with those recorded in Measurement Recorder. Recall that participants in the Shared Experiments were instructed to write down definitions of the characters on paper before using Measurement Recorder. In this analysis, the words used in their paper-based definitions (i.e. words filled in ‘from’, ‘to’, ‘include’, ‘exclude’ and ‘at’ fields) for all four tasks were compared with those they recorded in Measurement Recorder. All words were converted to lowercase. Numbers, punctuation and common stop words, such as ‘of’ and ‘a’, were removed. Unique words and unique definitions from paper versus computer-based sessions were then counted.

**Figure 12.**
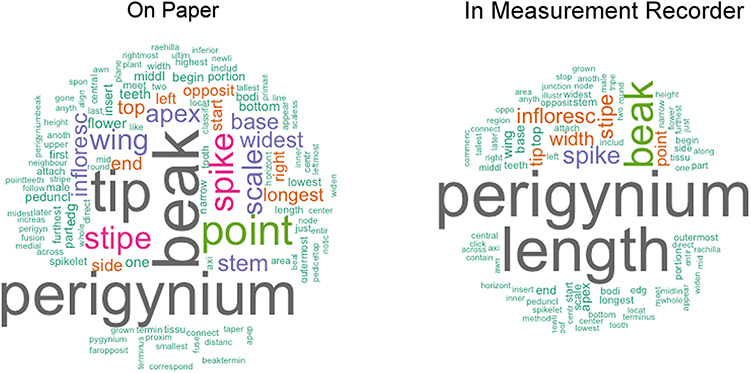
Word clouds generated using the definitions entered on paper versus those recorded in Measurement Recorder in Shared Experiment. The word clouds show that using Measurement Recorder reduces the number of unique content words used in character definitions.

Figure 12 presents the unique words used in the paper-based and computer-based session as word clouds. Visually, the cloud from the computer-based session is noticeably smaller than the one from the paper-based session. If presented as a bar chart with the term frequency sorted from high to low, the term distribution of the computer-based definitions would show a steeper curve and a shorter ‘tail’ (i.e. smaller number of low-frequency terms) than that generated from the paper-based definitions. This difference indicates that shared access to character definitions in Measurement Recorder help reduce term and definition variation and promote convergence on character definitions. Specifically, the definitions in the paper-based session contained 118 unique words, while those in the computer-based session contained 89, and that was a 25% reduction in unique word count in the computer-based definitions. Collectively, considering the words filled in ‘from’, ‘to’, ‘include’, ‘exclude’ and ‘at’, the paper-based session generated 122 unique definitions, while the computer-based session generated 64 unique definitions, showing a reduction of 48% unique definitions in the computer-based session.

From user response and from the quantitative evidence presented above, it is clear that character sharing and reusing features of Measurement Recorder promotes definition convergence among users.

### Research Question 5: Does shared access to measurement characters result in users accidentally using a character not intended?

To answer this question, we analyzed the scientific correctness of the characters created by the shared-NS and shared-ES participants. In the analyses below, attempts were made to differentiate the mistakes caused by not having sufficient biology knowledge from the mistakes caused by poor system design.

First, participants’ familiarity with the biological terms in the task illustrations was assessed based on their answers to the question ‘I am familiar with the terms (labels) in task illustration’, as shown in Table 7. These data show that participants in the shared-NS group possess a lower level of knowledge than those in the shared-ES group. This difference is statistically significant (Fisher Exact Test, *P*-value < 0.001).

**Table 7.** Participants’ familiarity with the biological terms in task illustrations

Question		<25%	25–50%	51–75%	76–100%
I am familiar with the terms (labels) in task illustrations	Shared-NS (N = 14)	4	7	1	2
	Shared-ES (N = 18)	0	0	4	14

#### Quality of paper-based characters

Two botanists (co-authors) reviewed the characters participants created on paper. They were instructed to mark a character as ‘correct’ if the character definition would lead them to a measurement character that was the same as depicted in the task illustration. If the character definition did not make sense, described a different character or could be interpreted in multiple ways, they would mark the definition as ‘wrong’. They reviewed 10% of the total characters together to practice the review criterion and then each reviewed half of the remaining characters in the same room concurrently, where they carried out limited discussion on some borderline cases. Paper-based character quality results are presented in Table 8.

**Table 8.** Errors found in paper-based definitions in the shared-NS and shared-ES groups

Task	Shared-NS total participants = 13, total characters = 52		Shared-ES total participants = 19, total characters = 76	
	Wrong character count	Percentage	Wrong character count	Percentage
Task 1	5	10%	4	5%
Task 2	7	14%	5	7%
Task 3	7	14%	8	10%
Task 4	10	20%	8	10%
Total	29	56%	25	33%

Table 8 shows that the shared-ES group made fewer errors (error rate = 33%) than the shared-NS group (error rate = 56%). This difference is statistically significant (two-proportion Z-test, *P* < 0.05), confirming the two groups possess different levels of knowledge on the task characters. Based on the number of errors made on different tasks, Task 1 is the easiest and Task 4 is the most difficult, especially for the shared-NS group.

#### Quality of machine-based characters

Errors in machine-based characters were identified by examining the user logs of the Shared Experiment, task by task, for the shared-NS and shared-ES groups. Measurement characters created by participants with either an incorrect definition or nonmatching unit were counted as errors and categorized as wrong definitions and/or wrong units, indicated in Table 9 with subscripts ‘d’ and/or ‘u’. It was not considered an error when participants chose not to reuse any available character and created a new, correct character. Users sometimes changed their initial decisions, for example, some first chose to ‘Use this’, then removed the character and selected ‘C&E’. In such situations, the error rate was assessed based on the final characters. Table 9 records the errors in each category (‘d’ vs. ‘u’) by methods, groups, and tasks.

**Table 9.** Errors found in machine-based definitions in the shared-NS and shared-ES groups

Task	Method						Group (N)
	Use This		C&E		Create New		
	Correct	Error	Correct	Error	Correct	Error	
Task1: Preloaded character defined with an illustration, but does not match the task	NA	0	7	0	6	0	NS (13)
	NA	2_d_	11	0	6	0	ES (19)
Task2: Preloaded character defined with text definition, and matches the task on definition and unit.	7	N/A	2	1_d_	1	2_d_	NS (13)
	13	N/A	4	1_d_	1	0	ES (19)
Task3: Preloaded character defined with text definition, and matches the task on a definition, but not on the unit.	NA	3_u_	3	3_d_ + 1_u_ + 2_u, d_	0	1_d_	NS (13)
	NA	7_u_	6	2_u_	4	0	ES (19)
Task4: All three preloaded characters use illustrations as definition, but none matches the task	NA	5_d_	0	2_d_	3	2_d_	NS (13)
	NA	9_d_	2	4_d_	3	1_d_	ES (19)
Total	7	8	12	9	10	5	NS (13)
	13	18	23	7	14	1	ES (19)

Subscripts ‘d’ and ‘u’ indicate error types: ‘d’ indicates errors in the measurement method definition, and ‘u’ indicates errors in the unit selected.

The vast majority of the participants reviewed the preloaded characters, before deciding on the next action (‘Use this’, C&E or create a new character). Two exceptions were participants ‘ns07’, who never checked any character on any task and went straight to create her/his own character, and ‘es11’, who did not check any available characters for Task 3 and Task 4. The interactions recorded in the user log show that all users from both shared-NS and shared-ES groups used all of the three different methods, except for ‘ns07’.

Table 9 shows that in the majority of the cases, participants correctly recognized whether a preloaded character matched the task on hand. The preloaded character for Task 1 did not match the task, and 94% of participants correctly rejected that character. Only 2 out of 32 chose ‘Use this’ and provided wrong definitions (‘2_d_’ in Table 9, Task1) while 18 selected C&E and 12 selected ‘Create New’ and created good characters. When the preloaded character matched the task (i.e. Task 2), 88% of participants chose either ‘Use this’ or C&E. On Task 3, where preloaded method definition matched the task, but the unit did not, participants correctly recognized the matching definition (84% ‘Use this’ or C&E), but around 50% failed to check and correct the unit for the task. This discovery has led to a feature redesign (see Software improvements below).

Task 4 had three distracting characters with similar names preloaded, but none matched the task on hand. Two of the distracting characters were defined with an illustration and one with a text definition, as shown in Table 10. Analyzing the results on this task reveals that illustrations alone were not sufficient for a user to decide if a given character was a good match. Close to half of the participants in either group decided definition (a) in Table 10 matched the task character based on the illustration. This is not correct based on taxonomic practice, because the landmark used in definition (a) (the bend connecting the perigynium body with the beak) is different from the landmark an expert would use for the task (i.e. ‘end of the wings’). However, undergraduate students, without being exposed to diverse species of *Carex*, would think these are similar because both measured to the tip of the perigynium. This observation suggests the need for both illustrations and textual definitions for measurement characters. When a measurement method is expressed textually, it allows the user to assess to what extent the method can be applied or generalized for unseen tasks. While illustrations are more visual and can depict features that are hard to put in words precisely and concisely, they offer little clue in terms of how to apply it beyond the exact same use case. The preloaded illustration and task illustration used in Task 1 were exactly the same with different measurement marks and that made it easy for participants to decide that the preloaded character was not a match. However, when the situation was more realistic and more difficult to decide, both shared-NS and shared-ES groups made mistakes.

When participants recognized a matching character, some chose to use the character as is (‘Use this’), while a smaller number of users chose to enhance the character by editing its method definition (C&E). Upon reviewing the user-provided definition on paper and in Measurement Recorder, we found that preloaded definitions provided by botanists were often more concise, but not very detailed (e.g. Task 2 length of inflorescence: measured from insertion of lowest scale to apex of inflorescence). Participants made efforts to enhance the definition by providing more details (e.g. Task 2 length of inflorescence: measured ‘from tip of male flower of the spike to bottom portion of the scale exclude any portion of the stem <at the very narrow point of the tip>’); however, due to insufficient domain knowledge, they sometimes introduced errors or ambiguity in the definitions. This was seen more often in the shared-NS group than in the shared-ES group. The shared-NS group enhanced a total of 21 characters with 9 wrong definitions, resulting in an error rate of 43%, while the shared-ES group’s error rate was 23% (=7/30, Table 9). Such errors are generated by a lack of domain knowledge and not due to software design. Professional botanists and botany students, the target users of Measurement Recorder, are less likely to make such mistakes. However, the software should provide adequate error prevention mechanisms whenever possible, for example, by allowing the users to verify and confirm their choices (see Software improvements below). In addition, we recognize that incremental enhancements may lead to concept drifting, resulting in a character that is semantically different from the initial character. Measurement Recorder is unable to prevent drifting. In this experiment, we did not see evidence of concept drifting, but this phenomenon should be monitored and further studied to assess the trade-off between the benefit of improved definitions and the risk of concept drifting.

In the computer-based definitions, the shared-NS group created 22 (= 8 + 9 + 5) characters with errors, compared with the error count of 29 in the paper-based definitions (see last row in Table 9). The shared-ES group’s errors did not reduce from paper-based (25) to computer-based (26 = 18 + 7 + 1). However, if the design issue with units was discounted (as it has been fixed in the redesign), then the errors would be reduced to 18 (=22-4) from 29 for the shared-NS group, and reduced to 17 (=26-9) from 25 for the shared-ES group. This suggests that the use of Measurement Recorder has the potential to reduce errors and improve character definition quality.

To summarize, error analyses of paper-based and computer-based exercises suggest that the software supports participant recognition of a matching character. A noticeable number of errors introduced by choosing the wrong unit have been addressed in the software. The vast majority of errors were due to participants’ lack of sufficient biological knowledge. The findings suggest that using Measurement Recorder can help reduce the latter type of errors.

## Software improvements

In addition to the software issues identified above, an open-ended question in the postexperiment questionnaires allowed participants to suggest improvements. Many of these have been implemented in Measurement Recorder and the Character Recorder in which Measurement Recorder is a module. All of these changes have been approved by the three botanists on the project.

**Figure 13.**
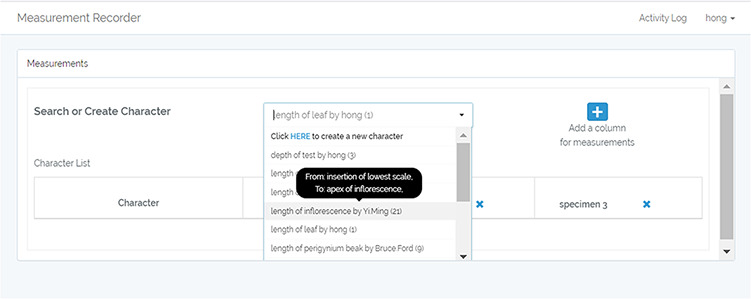
New feature: ‘Tooltip’ in Measurement Recorder allows users to quickly review relevant character for reuse.

**Figure 14.**
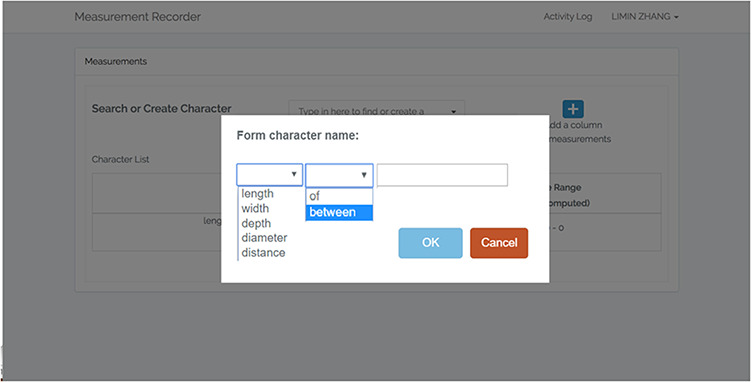
New feature: Character formation dialog in Measurement Recorder to ensure consistency in character naming with of/between (length of leaf is accepted, but not leaf length).

- When characters accumulate, it can become difficult to select relevant ones to reuse. To address this issue, tool-tips have been added in the character search/dropdown selection (Figure 13). Mousing-over a character triggers the display of the character’s verbal or visual definition.
- To bring users’ attention to the unit information, a mechanism has been added to send the user to the unit section automatically after they fill out the definition form.
- As an error prevention mechanism, and to further address the unit issue, a series of confirmation dialogs have been added for the user to review both the definition and the unit before saving a character when they ‘Use this’ or ‘C&E’ a character.
- A simple form for forming character names has been put in place (Figure 14). To maintain consistency in character naming, the form of ‘“character” of/between “structure”’ is adopted in Measurement Recorder, and the form eliminates character naming errors such as ‘leaf length’ as the correct form is ‘length of leaf’.
- A set of labels have been changed. For example, the new label for the search/create character box is now ‘Search or Create Character’ (Figure 13).

## Discussion

Data collected from our two usability experiments has shown that Measurement Recorder enables authors to express the semantics of the characters they use in an efficient and user-friendly manner. Furthermore, it accepts all characters and definitions associated with newly defined characters and allows addition of terms as needed to the ontology. Thus, in addition to a measurement recording tool, Measurement Recorder serves as a knowledge acquisition layer that passes knowledge about characters from authors to the ontology (or ontologies), making these accessible to ontology engineers.

Under the influence of the OBO Foundry (27), many ontologies in biology domains are developed in a ‘controlled’ manner, where the inclusion of terms and their relationships is decided by a small group of ontology engineers with or without deep domain knowledge. User-suggested new terms are vetted through a lengthy decision process before addition.

This practice is at odds with the basic principle of the Semantic Web, whose motto is to allow ‘Anyone to say Anything about Any topic’ (the AAA principle). The Semantic Web suite of technologies, Resource Description Framework (RDF) (https://www.w3.org/RDF/), RDF Schema (https://www.w3.org/TR/rdf-schema/), and OWL Ontology Language (https://www.w3.org/OWL/), provide tools to reconcile different facts and opinions (28). We do not believe our authors would need approval or should wait for weeks to add terms to ontology. Our approach is to encourage authors to utilize ontologies and through frequent use and improve the quality of the ontologies and the phenotypic data. At this time, we do not know if giving authors direct access to ontologies would lead to high disagreements in data or less effective RDF data stores. In fact, frequency of term/character usage should be a good measure of whether they deserve a spot in ontology, and the disagreements in the data stores would expose the issues we have in the ontologies or data. Once characters are linked to ontologies via tools such as Measurement Recorder, inappropriate usage (‘conflicts’) can be identified through logic reasoning and/or RDF shape checking over the ontology and the data. One goal of our next user studies on Character Recorder is to collect evidence in this regard. Recently, the idea of allowing authors to express semantic relationships, and expose potential conflicts, has been adopted by others, for example in the Gene Ontology (29).

In our design, new structure terms and their definitions are added under the class ‘new additions’ and receive their permanent IDs in the ontology right away. Terms under ‘new additions’ may become a subclass of other classes later at the discretion of an ontology engineer to support appropriate semantic modeling of relevant concepts. New characters are added under Quality (equivalent to PATO: quality) > Perceived quality and are associated with the structure (e.g. leaf) they measure. There is often a set of standard characters agreed upon by a community that should be included in the ontology. Variations in these characters created by users via Measurement Recorder, or other similar tools, can be added as subclasses or taxon-specific variations of pertinent standard characters. It is highly possible that the terms contributed by different users are merely different expressions of the same concepts. Based on definitions (including the term, its verbal definition, a sample sentence and a taxon example), these terms can be associated as equivalent classes if appropriate. Much of this association work will be carried out manually by an ontology engineer, given user-provided information and evidence of usages. For example, when character usages converge to a certain set of characters (community approved), rarely used ones can become ‘deprecated’ or ‘obsoleted’ (meaning the term should no longer be used), and past usages can be associated with the community approved characters as equivalent classes. For these cases, we prefer to use ‘equivalent class’ construct provided in OWL over any ‘synonym’ annotations (exact synonyms, broader/narrower synonyms, related synonyms) that are widely used in traditional thesauri and some ontologies, because a synonym annotation is not a formal semantic construct, and cannot be used in logic reasoning. While the ontology engineer is moving and associating terms, users can use their new terms in their descriptions or matrices. The associations established by the ontology engineer will only add to the semantics of those terms Part of the ‘Authors’ project is to assess the feasibility of this approach and the expected workload of the ontology engineer.

## Related work

The difficulty in relating scientific findings in published works has long been recognized, for example, in ‘Undiscovered Public Knowledge’ (30) before the Web era. The need to connect and compare findings in biological science publications has only become more urgent since then. It has led to massive curation and databasing of information content (see e.g. (2)) and broader semantic modeling proposals (31). All these efforts are to support the FAIR data principles (Findable, Accessible, Interoperable, Reusable) (32) and to make data useful for large scale computerized analyses.

Several annotation software platforms have been developed, such as Phenex (33) and Deomeo (34), but these are different from Measurement Recorder. Phenex helps curators convert ‘already published’ phenotype descriptions to a semantic format such as EQ formalism (2). Deomeo allows curators to annotate a broader range of web scientific documents using ontologies. Measurement Recorder, on the other hand, helps authors compose ‘new’ descriptions in a semantically clear manner to reduce the need for downstream conversion/annotation. Curators are trained in using and constructing ontologies, but are not necessarily an authority in the subject matter. Features tailored for highly trained curators are often difficult to use for phenotype authors in general. The goal of Measurement Recorder and related tools is to produce semantically clear descriptions for publication so that data can be harvested directly without intercurator variations. Conceptually, this is achieved by making sure the software tools are truly geared toward the needs of the authors in documenting characters and writing phenotypic descriptions. Such authors may never use ‘computable characters’ in their own research, but they may be willing to adopt ‘the tool’ because it benefits them in other ways. For example, using a standard set of characters speeds up the writing process or it allows their data to be used more seamlessly by other researchers.

While professional curation is aided by natural language processing (e.g. information extraction, named entity recognition) and document retrieval techniques, given the intractable publication volumes and intrinsic difficulties in professional curation, curators alone cannot ‘move the mountain’ that is ever growing (1, 35, 36). Alternative curation strategies have been proposed and used: crowd-sourcing (37, 38) (see (39) on strategies of using crowd-sourced data that are of lower quality in general) and curation involving authors (35, 36). In an experiment, the journal FEBS Letters requested consenting authors to submit a structured abstract, containing the identifiers of interacting proteins, their interaction types and the experimental method used. Their findings show that author contributions were of good quality and useful (36). A strategy used by FlyBase to prioritize articles for professional curation was to automatically email authors and ask them to list the genes studied and the data types described in their newly published papers. They found the strategy effective if authors were emailed between 0 to 2 months following the publication of their article (35). Other author curation exercises include the TAIR project that allowed authors to submit Gene Ontology term annotations on *Arabidopsis* genes (38); PomBase’s Canto (40) web application that provided a series of web forms for author curators to enter information such as genes mentioned in their articles, protein modifications, interactions, phenotypes and alleles for these genes and Pathway Commons’ plan for a collaborative workspace for pathway curation (41, 42).

Karp (38) considered the last 20 years of annotation experience of the bioinformatics community and commented on the crowd-sourcing and author curation approaches. He suggested ‘[t]he vast weight of empirical evidence to date suggests that crowd-sourced curation is not a successful model for biological databases’, and ‘the author–curation model’ shows more promise for boosting curator efficiency. However, its limitations include that the quality of author-submitted annotations is uncertain, the response rate is low (but significant), and to date author curation has involved relatively simple forms of annotation involving one or a few types of data. Furthermore, shifting curation to authors may simply redistribute costs rather than decreasing costs; author curation may in fact increase costs because of the overhead involved in having every curating author learn what professional curators know: curation conventions, curation software and curation procedures.”

All the author–curation projects reviewed above recrui-ted authors to share the workload of professional curators. Aside from the workload, in complicated phenotype curation, intercuration variation is a more serious obstacle that cannot be adequately addressed after an article is published. What we have proposed and are researching is enabling authors to embed semantics and relations while they are working on their data for a publication. We agree with the questions raised by Karp (38) and are investigating how to bring authors into semantic curation without making them ‘learn what professional curators know’ by leveraging the AAA principle of the Semantic Web techniques. A survey we conducted recently shows that authors are willing to put in additional efforts to produce semantic data (publication in preparation). Our research strives to create effective, yet easy to use software to support this goal.

Different from involving authors in postpublication curation, our approach adds structure (including semantics) to biological data at the time of the publication. This approach is less recognized, probably because related projects often fail to gain broad support from publishers and related communities. One recent example is the shutting down of the Digital Protologue Database website in September 2019 (43, 44). This site has been in use since 2017 accumulating digitized protologs for the journals Systematic and Applied Microbiology (SAM) and Anton van Leeuwenhoek. Recently, SAM made the submission of the digital protolog via a Web form compulsory for any new description. SAM’s requirement was very similar to the experiment conducted by FEBS Letters (36). Aside from the lack of funding, little is known about authors’ response to SAM’s form-based digital protolog interface and what lessons future endeavors could learn from this effort. Forcing authors to use a limited set of terms was cited as one of the main reasons that Flora of North America management abandoned their structured input form for taxonomic descriptions over 15 years ago (B.A. Ford, personal communication, 2018, J.A., Macklin, personal communication, 2011). For this reason, we give authors freedom to express their data in our design of the tools. We believe semantic publication is the long-term solution to the problem of making biological data FAIR and call for more research in this area.

Several innovative biodiversity phenotype information management platforms are currently being developed in the semantic publication area, for example, the taxonomist’s workspace metaphor used in TaxonWorks (45) and ontology-driven configurable data forms used in Morph∙D∙Base (46). Both are backed by ontologies to varied extends. The key differences between these two platforms and Measurement Recorder (and Character Recorder) include (i) new metaphors/data input forms are adopted in the former, while Measurement Recorder stays with the traditional spreadsheet, and (ii) users cannot directly add terms and definitions to the ontologies in the former, in contrast, in Measurement Recorder, user terms and definitions are added directly to the ontologies. Terms may be modified or deprecated only if they were found to create conflicts during use. Conflict identification and resolution by authors are features of Character Recorder and a mobile application that are not reported here. The three projects are exploring a new territory and can benefit from the lessons learned by one another. For example, the ‘green wheel’ employed in Measurement Recorder (Figure 10) was inspired by the radial button first created by the TaxonWorks group. Participants in our experiments commented favorably on this visual feature.

Although there are sophisticated measurement ontologies being proposed that share the key idea of semantically documenting measurements for various domains (47–49), we are not aware of any publicly accessible platform that allows authors to describe how a measurement is taken and supports authors adding their descriptive terms to phenotype ontology.

## Conclusion and future work

In this paper, we emphasize the need for a software platform that helps authors semantically describe their phenotype data to (i) curtail intercurator variation in curated data and to (ii) help authors produce semantically clear data for computation use. We describe a software prototype, Measurement Recorder, and report a set of usability studies with three different user groups possessing different levels of domain knowledge. A now slightly dated version of Measurement Recorder is running at http://shark.sbs.arizona.edu/mr/individual/public/. The source code (JavaScript) and the ReadMe file of Measurement Recorder are publicly accessible at https://github.com/biosemantics/Measurement-Recorder-Individual-Site. The backend of Measurement Recorder is supported by a set of APIs that manipulate the ontology. The source code and ReadMe of the ontology API endpoints are available at https://github.com/biosemantics/charaparser-web.

The results obtained from questionnaires and user interaction log data suggest that users can use Measurement Recorder without training and find it easy after the initial learning curve. Users also appreciate the semantic features that enhance data quality. Measurement Recorder’s character reuse features help character convergence by 48% and have the potential to reduce errors in user-created characters. The results also show that participants with more biological knowledge favor Measurement Recorder more than participants with less domain knowledge.

Based on the lessons learned from this study, we have already started to implement a Character Recorder prototype platform, where both categorical and continuous characters are supported. Measurement Recorder has become an integral part of this platform. The set of semantic model patterns developed by Lars Vogt and other colleagues for organism phenotypic knowledge (50) will be used in this project to represent the semantic data recorded by the authors as an RDF knowledge graph, in addition to publishable narratives. In the near future, we will conduct experiments on the Character Recorder with a number of *Carex* experts to (i) assess the usability of the entire system, (ii) characterize and quantify the potential conflicts that may result from terms added by different authors, (iii) evaluate the effectiveness of the conflict resolution mechanism (in development) and (iv) examine the division of work between authors as domain knowledge providers and ontology engineers as knowledge formalizers.

## APPENDIX 1. Preloaded Characters

Preloaded characters:

**Table:**

Number of illustrations	Number of usage	Character creator	Character name
2, include beak or stipe. not match task	20	Bruce Ford	Length of perigynium

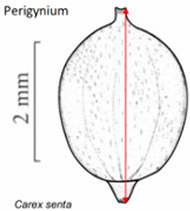
			
1, no match task	3	Bruce Ford	Length of perigynium beak

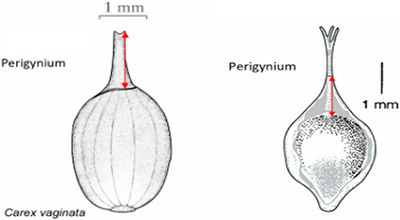
			
No, def not match	2	Bruce Ford	Length of winged perigynium beak

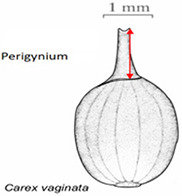
			

**Table:**

Number of illustrations	Number of usage	Character creator	Character name
1, not match task	2	Yi Ming	Length of perigynium beak

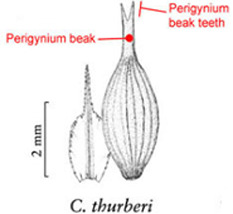
			
No illustration, def not match	20	Yi Ming	Width of spike

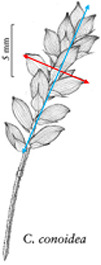
			
No illustration, def match	3	Yi Ming	Length of inflorescence

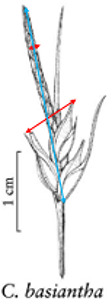
			

## APPENDIX 2. Individual Experiment Materials

**Subject ID:___________**

### Instruction to Measurement Recorder for Data Quality Control Experiment

**Please read all the instructions carefully before start.**

**Task:** Students in your class are given an assignment to define a set of characters for some objects and record their measurement values. A **character** is a physical property for an object (for example, ‘height of tree’ for a tree). The measurements you recorded will be compiled into a database for comparative studies, along with your character definitions. Character definitions are needed because it can be hard to compare measurement values without knowing how the characters were measured.

In this experiment, you will complete the following, check the box when you’ve completed a step:

□ Sign the consent form. There are two forms, please sign both. Keep one copy for yourself.
□ Follow the tabs on your browser:

□ Watch the video (https://www.screencast.com/t/c0dZCVZI), which is shown on the first tab in your browser.
□ On the next tab, use the Measurement Recorder to define and record measurements for four objects **in the order that is given.** You will define all four characters. ‘Ignore’ characters created by others if they show up. If encountering questions, do what you think makes sense. Lab assistant should be called when there is a software malfunction.
□ Create character 1 in Measurement Recorder, and enter the measurement given.
□ Create character 2 in Measurement Recorder, and enter the measurement given.
□ Create character 3 in Measurement Recorder, and enter the measurement given.
□ Create character 4 in Measurement Recorder, and enter the measurement given.

□ On the next tab, please fill out the questionnaire at https://uarizona.co1.qualtrics.com/jfe/form/SV_0lk6ZvCH81ba0oB.

□ Proceed to receive your payment.

### Task Sheet

**Instruction:**

The blue marks in the picture indicate how a character about the leaf is measured. Create/record the character and the measurement indicated using the software.

### Task Sheet

**Instruction:**

The blue marks in the picture indicate how a character about the leaf is measured. Create/record the character and the measurement indicated using the software.

### Task Sheet

**Instruction:**

The blue marks in the picture indicate how a character about the cell phone is measured. Create/record the character and the measurement indicated using the software.

### Task Sheet

**Instruction:**

The blue marks in the picture indicate how a character about the eyes is measured. Create/record the character and the measurement indicated using the software.

## APPENDIX 3. Shared Experiment Materials

**Subject ID:___________**

### Instruction to ‘Measurement Recorder for Data Quality Control Experiment’

**Please follow all of the instructions carefully**

Thank you for participating in our experiment! Your contribution is critical to our development of software that will allow independent researchers to write comparable morphological descriptions of plants that computers can ‘understand’. This will ultimately allow biologists to generate large dataset that can be used for broad studies related to the taxonomy and classification of all plants.

**First check** and make sure that you have an informed consent sheet, four task sheets, and some yellow post-it notes on your desk. If you encounter a software issue, please raise your hand for help. Stick a yellow note on your computer when you have completed the experiment.

□ Sign the consent form. Dr Cui will collect the signed form, along with the completed task sheets at the end of the experiment.
□ Go to **https://tinyurl.com/y7e6hmst** and watch the introduction video.

Your assignment is to collect measurement data on some sample images of *Carex* characters (for example, perigynium). The measurements that you will record in the ‘Measurement Recorder’ software will be compiled into a database for comparative studies, along with character definitions. Character definitions are needed because it can be hard to compare measurement values without knowing how the characters were measured. Characters created and used in the past by others are available in the system. If an existing character matches your task, you should use the character. If you do not find such a character, you should create a new character to record the value.

1. For the first step, fill out the hard copy definition forms for all four tasks, in the order given.
2. In Chrome, go to http://shark.sbs.arizona.edu/mr/individual/ and use the ‘Measurement Recorder’ to complete the second step for all four tasks, in the order given. **To login, use the email address**  yoursubjectid@test.com (your subject id is found at the top right corner of this instruction sheet) **password: 00000000 (which is 8 zeros)**.
  - You will find some characters are already defined in the software and available for you to use.
    1. When you select an existing character, there are two options available:
      - The ‘Use this’ button allows you to use this character as is without editing its definition.
      - The ‘Clone and enhance’ button allows you to enhance the character’s definition to make it more complete or more understandable. This allows you to improve the definition without changing the meaning of the original definition.
  - You can also create a new character, define it (i.e. explain how the character should be measured) and record the value.
  - You will record all values under the **measurements** column in ‘Measurement Recorder’.
3. Please fill out the questionnaire at **https://tinyurl.com/ycgr9e94**.
4. When you have completed the tasks and the questionnaire, keep your browser open and stick a yellow post-it note on your computer. Dr Cui will come to you to finalize the experiment and give you your honorarium.

**Thank you for participating in this experiment!**

**Screenshots of Measurement Recorder for Your Reference**

### Task Sheet 1 for Subject ID:______________________

**Instruction:**

The blue marks in the picture indicate how a character ‘length of perigynium’ is measured.

1. Fill out the form below to describe how the character is measured as it is depicted in the drawing.
2. In the Measurement Recorder, select an appropriate character that matches the character depicted. Enter the measurement value indicated. If you do not find a matching character, feel free to create and define a new character.

Fill in the fields (boxes) that are ‘relevant’ to this character. Note that you may not need to fill in all of the fields.

**Table:**

Character name:	Length of perigynium
**Measured from:**	
**To:**	
**Include:**	
**Exclude:**	
**At:**	
**(e.g. at the widest point)**	

Plan B: You will be informed if you need to use anything on this side.

Character name: length of perigynium

□ Use the existing character #___________________
□ Clone and enhance the existing character #____________________

○ Enhancements made:

□ Create my character. Put ‘same’ in a field if it does not change from your initial entry. Otherwise, enter a new entry to the ‘relevant’ fields below.
  **Table:**
  Character name:	Length of perigynium
  **Measured from:**	
  **To:**	
  **Include:**	
  **Exclude:**	
  **At:**	
  **(e.g. at the widest point)**	

### Task Sheet 2 for Subject ID: ________________________

**Instruction:**

Repeat the same steps you followed on Sheet 1 for the character depicted below.

Fill in the fields (boxes) that are ‘relevant’ to this character. Note that you may not need to fill in all of the fields.

**Table:**

Character name:	Length of inflorescence
**Measured from:**	
**To:**	
**Include:**	
**Exclude:**	
**At:**	
**(e.g. at the widest point)**	

Plan B: You will be informed if you need to use anything on this side.

Character name: length of inflorescence

□ Use the existing character #___________________
□ Clone and Enhance the existing character #____________________

○ Enhancements made:

□ Create my character. Put ‘same’ in a field if it does not change from your initial entry. Otherwise, enter a new entry to the ‘relevant’ fields below.
  **Table:**
  Character name:	Length of perigynium
  **Measured from:**	
  **To:**	
  **Include:**	
  **Exclude:**	
  **At:**	
  **(e.g. at the widest point)**	

### Task Sheet 3 for Subject ID:______________________

**Instruction:**

Repeat the same steps you followed on Sheet 1 for the character depicted below.

Fill in the fields (boxes) that are ‘relevant’ to this character. Note that you may not need to fill in all of the fields.

**Table:**

Character name:	Width of spike
**Measured from:**	
**To:**	
**Include:**	
**Exclude:**	
**At:**	
**(e.g. at the widest point)**	

Plan B: You will be informed if you need to use anything on this side.

Character name: width of spike

□ Use the existing character #___________________
□ Clone and Enhance the existing character #____________________

○ Enhancements made:

□ Create my character. Put ‘same’ in a field if it does not change from your initial entry. Otherwise, enter a new entry to the ‘relevant’ fields below.
  **Table:**
  Character name:	Width of spike
  **Measured from:**	
  **To:**	
  **Include:**	
  **Exclude:**	
  **At:**	
  **(e.g. at the widest point)**	

### Task Sheet 4 for Subject ID:_______________________

**Instruction:**

Repeat the steps as the first character for the character depicted below.

Fill in the fields (boxes) that are ‘relevant’ to this character. Note that you may not need to fill in all of the fields.

**Table:**

Character name:	Length of perigynium beak
**Measured from:**	
**To:**	
**Include:**	
**Exclude:**	
**At:**	
**(e.g. at the widest point)**	

Plan B: You will be informed if you need to use anything on this side.

Character name: length of perigynium beak

□ Use the existing character #___________________
□ Clone and Enhance the existing character #____________________

○ Enhancements made:

□ Create my character. Put ‘same’ in a field if it does not change from your initial entry. Otherwise, enter a new entry to the ‘relevant’ fields below.
  **Table:**
  Character name:	Length of perigynium
  **Measured from:**	
  **To:**	
  **Include:**	
  **Exclude:**	
  **At:**	
  **(e.g. at the widest point)**	
